# Radiation-induced cavernous malformation within a vestibular schwannoma: A case report

**DOI:** 10.1177/2050313X251390626

**Published:** 2025-10-28

**Authors:** Alex Z. Graboyes, Alexandra E. Quimby, Sabrina Heman-Ackah, Sara L. Stone, Jason A. Brant, Douglas C. Bigelow, John Y. K. Lee

**Affiliations:** 1 Department of Otorhinolaryngology – Head & Neck Surgery, University of Pennsylvania, Philadelphia, PA, USA; 2 Department of Otorhinolaryngology – Head & Neck Surgery, Dalhousie University, Halifax, NS, Canada; 3 Department of Neurosurgery, University of Pennsylvania, Philadelphia, PA, USA; 4 Department of Pathology and Laboratory Medicine, University of Michigan, Ann Arbor, MI, USA; 5 Division of Otolaryngology – Head and Neck Surgery, Department of Surgery, University of Wisconsin School of Medicine and Public Health, Madison, WI, USA; 6 William S. Middleton Memorial Veterans Hospital, Madison, WI, USA

**Keywords:** cavernous malformation, cavernoma, radiation, radiotherapy, vestibular schwannoma, acoustic Neuroma

## Abstract

Cavernous malformations are a well-described complication of intracranial radiation; however, have only once previously been described within a vestibular schwannoma following radiotherapy. We report a case of a 78-year-old woman presenting with new hemifacial spasm 16-years following fractionated radiotherapy for a vestibular schwannoma, with imaging suggesting the formation of a cavernous malformation within the tumor. The patient underwent translabyrinthine resection, with final pathology confirming the diagnosis of a cavernous malformation within the vestibular schwannoma. This case highlights the need to maintain a broad differential in patients with new symptoms following radiotherapy for vestibular schwannomas, including hemifacial spasm. Cavernous malformations should be considered among potential radiation-related complications in patients with vestibular schwannoma.

**Level of Evidence:** IV

## Introduction

Vestibular schwannomas (VS) are benign tumors of the internal auditory canal (IAC) and cerebellopontine angle (CPA) originating from Schwann cells of the vestibulocochlear nerve. Common management strategies for these tumors include surgery, stereotactic radiotherapy (SRT), and surveillance, with the best-suited option depending on a variety of patient and tumor factors. SRT has been well demonstrated to be a safe and effective treatment for small- and medium-sized tumors.

Cavernous malformations are benign vascular malformations that rarely arise post-SRT. These malformations are best visualized with T2-weighted magnetic resonance imaging (MRI).^1^ However, there has been only two prior cases describing a cavernous malformation arising within a VS following SRT.^2,3^

We report a case of a suspected radiation-induced cavernous malformation developing within a VS previously treated with SRT.

## Case report

A 78-year-old female presented to clinic with new symptoms of hemifacial spasm and progressive gait ataxia, as well as unsteadiness and imbalance; her history was significant for having been treated for a right-sided VS with a 6-week course of fractionated SRT, due to being treated at an institution that did not have access SRT, in 2006. Her posttreatment course had been otherwise unremarkable aside from hearing loss on the right side, and her past medical and family history were otherwise noncontributory. Her otologic, cranial nerve, and remaining physical examination was unremarkable, aside from using a cane for ambulation. The patient described symptoms of hemifacial spasm occurring an average of four times per day. Each episode was described as beginning with right-sided facial twitching, followed by sustained contraction of the right mouth and eye, with subsequent relaxation.

**Figure 1.**
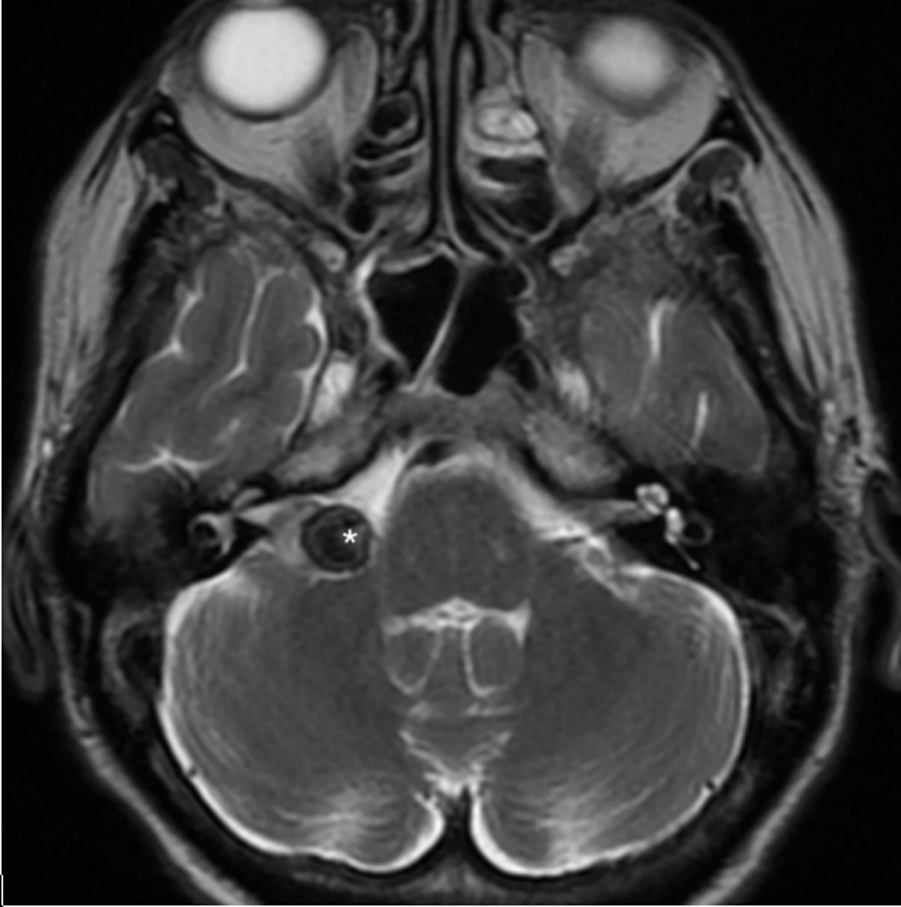
Axial cut of T2-weighted MRI demonstrating hypointense focus within right CPA lesion. MRI: magnetic resonance imaging.

In July 2022, the patient underwent contrast-enhanced MRI, which demonstrated a 1.3 × 1.7 × 1.1 cm enhancing mass in the right CPA extending into the IAC, consistent with the previously diagnosed and treated VS. Additionally, a secondary lesion was identified within the VS, which was hypointense on T2-weighted images. This was felt to be consistent with a possible cavernous malformation. Repeat imaging in February 2023 demonstrated the VS to be stable in size, but an increase in size of the T2-hypointense lesion, now measuring 1.1 cm in maximal plane (0.9 cm on previous imaging; Figure 1).

The hemifacial spasms were deemed to be most likely due to facial nerve irritation by the VS or cavernous malformation. The patient was evaluated by neurology and initiated treatment with oxcarbazepine with good effect, and her symptoms of hemifacial spasm subsided. However, due to the progressive increase in size of the lesion, its changing nature on imaging, and the desire for pathologic analysis, a decision was made to pursue surgical resection. The patient underwent translabyrinthine resection in May 2023. Preoperative embolization was not performed as this was a low-flow, angiographically occult lesion that was also lacking a significant arterial supply amenable to embolization

**Figure 2.**
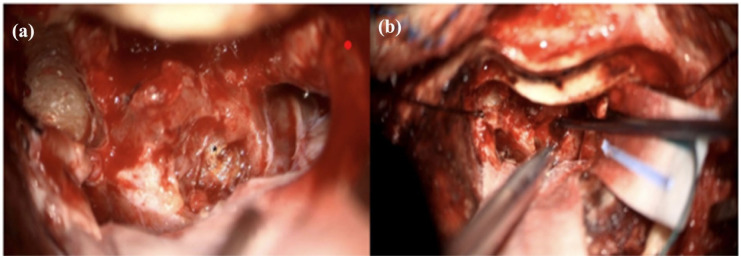
Intraoperative images demonstrating, (a) gross appearance of suspected cavernous malformation within the CPA portion of the VS; (b) old blood contents of the lesion upon probing. VS: vestibular schwannomas; CPA: cerebellopontine angle.

**Figure 3.**
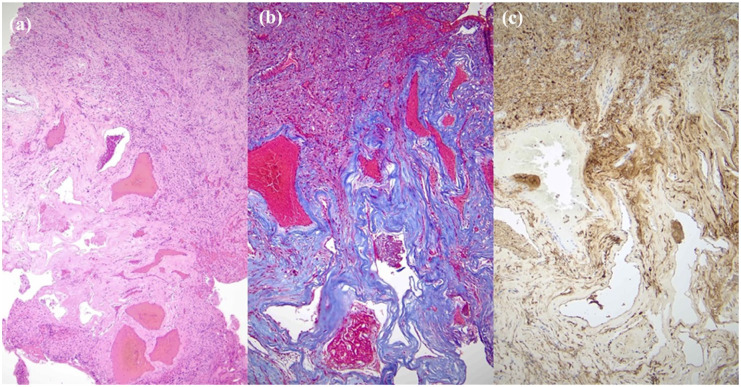
Microscopic appearance of the vascular malformation, (a) Hematoxylin and eosin (H&E) stained section showing tumor and adjacent vascular lesion (50×), (b) trichrome stained section highlighting hyalinized vessels and demonstrating absence of muscular wall (100×), (c) immunohistochemical stain for S100 highlights rare tumor cells between vessels (100×).

Intraoperatively, within the CPA portion of the lesion, a distinct lesion was noted which appeared in keeping with a cavernous malformation; it had a lobular appearance with hemosiderin-colored staining, and upon probing, contained both fresh and old-colored blood (Figure 2(a) and (b)). Pathologic evaluation revealed schwannoma with a focus of clustered, ectatic, thin-walled, and hyalinized vessels with scant intervening tumor cells in keeping with a cavernous malformation, as well as areas with recent and remote hemorrhage as evidenced by blood and hemosiderin deposition (Figure 3(a)–(c)). The patient was discharged from hospital to acute rehabilitation for balance, gait, and mobility training due to ongoing imbalance postoperatively. The patient has since done well postoperatively.

## Discussion

To our knowledge, this is the second reported case of a cavernous malformation forming within a VS following SRT, with the prior reported case describing the cavernous malformation within the wall of the irradiated VS.^2^ Radiation-induced cavernous malformations are rare, delayed complications of cranial irradiation, with the majority of cases in the literature being reported in pediatric patients following cranial irradiation at a mean of 9.2 years following treatment.^1,2^ The incidence of naturally occurring cavernous malformation is approximately 0.02%–0.53%; however, patients who receive radiotherapy have been shown to have a six-fold increased risk of their development compared to the general population.^1^

Two prior cases have been described in the literature. In the first case, Sasagawa et al. described a patient developing hemihyperesthesia and mild hemiparesis 10 years post-gamma knife radiosurgery, which was done after they could not due a complete resection due to bleeding.^3^ Imaging demonstrated a new heterogenous and “popcorn-like” mass in the CPA, appearing adjacent to the VS. The patient’s symptoms improved 10 days after admission with no surgical intervention.^3^ In the second case Nussbaum et al. described a patient who developed increasing facial numbness 11 years post-SRT; imaging demonstrated the lesion to be in proximity to the trigeminal nerve.^2^ This patient’s symptoms resolved after retrosigmoid resection of the lesion.

An alternative diagnostic consideration in this case is postradiation intratumoral hemorrhage within the VS. Intratumoral hemorrhage following radiation for VS is a rarely described complication. One series of 1902 patients with VS treated by SRT over a 35 year-period found a cumulative incidence of intratumoral hemorrhage of 0.26%. Intratumoral hemorrhage occurred at a range of 2–130 months after SRT.^4^ The patient’s presentation with facial spasm raises the possibility that rapid expansion secondary to intratumoral hemorrhage is an alternate diagnosis.

Most cases of hemifacial spasm are felt to be due to vascular compression of the facial nerve; however, the etiology is not always obvious. In medically treated cases, antiepileptic medications are often first-line. Hemifacial spasm is a rare complication of tumors of the CPA.^5^ A case series and literature review by S.H. Lee et al estimated that tumor compression may be the cause of hemifacial spasm in 0.3%–2.5% of cases.^5^ In a patient with a known or previously treated VS or other CPA tumor, the occurrence of new hemifacial spasm should prompt consideration of either tumor growth, transformation, or the appearance of a new lesion, as was the case in the report presented herein. Cavernous malformations are a rare radiation-related complication in the CPA, but should be considered among one’s differential diagnosis. In our case, the hemifacial spasm was well-controlled with medical management, though surgery was ultimately pursued due to the changing nature of the lesion.

## Conclusion

Cavernous malformations are a rare complication of radiotherapy for intracranial lesions, including those in the CPA. This case demonstrates one possible presentation of this pathology, being only the second-reported case of a radiation-induced cavernous malformation forming in a VS. The occurrence of new symptoms occurring in a patient previously treated by SRT for a CPA lesion—including new hemifacial spasm, as was seen in our case—should prompt consideration of this rare radiation-related complication. Intratumoral hemorrhage is an alternative consideration. Though medical management should be considered as first-line treatment in the case of hemifacial spasm, surgery may be pursued for enlarging or otherwise persistently symptomatic lesions.
